# Comparison of Different Commercial Nanopolystyrenes: Behavior in Exposure Media, Effects on Immune Function and Early Larval Development in the Model Bivalve *Mytilus galloprovincialis*

**DOI:** 10.3390/nano11123291

**Published:** 2021-12-04

**Authors:** Manon Auguste, Teresa Balbi, Angelica Miglioli, Stefano Alberti, Sonja Prandi, Riccardo Narizzano, Annalisa Salis, Gianluca Damonte, Laura Canesi

**Affiliations:** 1 Department of Environmental, Earth, and Life Sciences (DISTAV), University of Genoa, 16136 Genoa, Italy; teresa.balbi@unige.it (T.B.); angelica.miglioli@obs-vlfr.fr (A.M.); laura.canesi@unige.it (L.C.); 2 Department of Chemistry and Industrial Chemistry (DICCI), University of Genoa, 16136 Genoa, Italy; stefano.alberti@edu.unige.it; 3 Department Regional Laboratory, Sector Organic Chemistry and Physical Analysis, ARPAL, 16149 Genoa, Italy; sonja.prandi@arpal.liguria.it (S.P.); riccardo.narizzano@arpal.liguria.it (R.N.); 4 Department of Experimental Medicine (DIMES), University of Genoa, 16136 Genoa, Italy; Annalisa.Salis@unige.it (A.S.); gianluca.damonte@unige.it (G.D.)

**Keywords:** nanopolystyrene, size, charge, *Mytilus*, hemocytes, NP-protein corona, immune responses, larvae

## Abstract

In the absence of standard methods for the detection/quantification of nanoplastics (NPs) in environmental samples, commercial nanopolymers are utilized as proxies for toxicity testing and environmental risk assessment. In marine species, a considerable amount of data are now available on the effects of nanopolystyrene (PS-NPs) of different size/surface characteristics. In this work, amino modified PS-NPs (PS-NH_2_) (50 and 100 nm), purchased from two different companies, were compared in terms of behavior in exposure media and of biological responses, from molecular to organism level, in the model marine bivalve *Mytilus*. Different PS-NH_2_ showed distinct agglomeration and surface charge in artificial sea water (ASW) and hemolymph serum (HS). Differences in behavior were largely reflected by the effects on immune function in vitro and in vivo and on early larval development. Stronger effects were generally observed with PS-NH_2_ of smaller size, showing less agglomeration and higher positive charge in exposure media. Specific molecular interactions with HS components were investigated by the isolation and characterization of the NP-corona proteins. Data obtained in larvae demonstrate interference with the molecular mechanisms of shell biogenesis. Overall, different PS-NH_2_ can affect the key physiological functions of mussels at environmental concentrations (10 µg/L). However, detailed information on the commercial NPs utilized is required to compare their biological effects among laboratory experiments.

## 1. Introduction

Nanoplastics (NPs) are currently defined as particles produced from plastic degradation and manufacturing, presenting a colloidal behavior, within the size range of 1–1000 nm [1]. Although NPs are expected to be nearly ubiquitous in aquatic compartments, their presence in the marine environment has been detected so far only in the North Atlantic Gyre [2,3] and, more recently, in coastal environments [4]. Such a lack of evidence is related to the still open issue of analytical procedures for their determination and characterization [5]. The formation of NPs from larger plastic items has been experimentally demonstrated [6,7,8,9]. Consequently, several studies recently focused on the development of alternative top–down processes to obtain fragmented NPs that are polydisperse and with irregular shapes, which can be representative of those occurring in the environment ([10] and refs. quoted therein).

However, in the absence of standard methods for the routine detection and quantification of environmental concentrations of NPs, different commercial nanopolymers are utilized as proxies of NPs for toxicity testing and environmental risk assessment [11]. These NPs are synthesized with a bottom–up approach as monodisperse and spherical, thus mimicking environmental NPs on the basis of size and surface charges, but not of shape and homogeneity, and they have been used to provide an estimate of the behavior of NPs in different aquatic media [12,13] and their biological impact in aquatic species, marine in particular [14,15,16,17,18].

In several marine invertebrates, NPs have been shown to affect the digestive system, lowering food input for small organisms feeding on nano–micro range, molting, embryo development, immune responses, and pro-antioxidant processes (reviewed in [19]). All these impacts may lead to a loss in energy of the animals or in the population in the environment. Several NP types were used, with the most widely utilized being polystyrene nanoparticles (PS-NPs) of different sizes and surface characteristics (i.e., plain or labeled with different fluorochromes, surface modified with cationic and anionic groups).

Bivalve mollusks have been shown to represent significant targets for nanosized particles: as suspension-feeders, they have highly developed processes for the cellular internalization of nano- and micro-scale particles (endo- and phagocytosis), which are integral to key physiological functions such as intra-cellular digestion and cellular immunity [20,21].

The model marine bivalve *Mytilus* has proven useful in evaluating the impact of different types of nanomaterials, including model PS-NPs ([19] and refs. therein). In the Mediterranean mussel *M. galloprovincialis*, amino-modified 50 nm PS-NH_2_ (from Bangs Lab. Inc., Fishers, IN, USA), previously used for investigating nano-bio-interactions in human cells [22] and effects in sea urchin embryos [23], has been utilized in studying the biological interactions of NPs in different experimental settings in vitro and in vivo [19,24,25,26]. The results underlined how mussel immune function in adults and early development in larvae can represent significant targets for NPs.

In the last years, the increasing availability of commercial PS-NPs with different sizes and functionalization has partly contributed to the increasing number of publications on the potential impact of NPs on aquatic organisms, including bivalves [18,27]. However, this raises some concern on the possibility to compare data obtained utilizing commercial NPs with different sizes and/or surface modifications and/or obtained from different sources.

In this work, the effects of amino-modified PS-NPs (PS-NH_2_) of different sizes (50 and 100 nm) and purchased from two different companies, were evaluated in *M. galloprovincialis* in terms of behavior in different exposure media and of in vitro and in vivo effects on immune responses in adults and on early larval development.

## 2. Materials and Methods

### 2.1. PS-NP Characterization

Amino-modified (-NH_2_) polystyrene nanoparticles (PS-NPs) of two different nominal sizes (50 and 100 nm) were purchased from two companies: Bangs Laboratories, Inc. (Fishers, IN, USA) (PS_50_-B and PS_100_-B) and Sigma-Aldrich (Milan, Italy) (PS_50_-S and PS_100_-S). PS_50_-B primary particles and their behavior in different media (milliQ water (MQ), artificial seawater (ASW), and mussel hemolymph serum (HS)) were previously characterized [28,29]. For other types of PS-NPs, primary characterization was carried out by Field Emission Scan Electron microscopy (FESEM) (Zeiss, Sigma 300, Jena, Germany). PS-NPs suspended in MQ water were spread onto a 0.22 µm polycarbonate filter, left to dry, mounted on aluminum holders, and sputter-coated with chromium, using a precision etching coating system (Quorum Technologies, G150TS plus, Lewes, UK). The hydrodynamic diameter (Z-average), polydispersity index (PDI), and surface charge (zeta potential in mV) of particle suspensions (50 µg/mL) in different media (MQ, ASW, and HS) were determined by Dynamic Light Scattering (DLS) using a Zetasizer Nano ZS instrument (Malvern Panalytical, Malvern, UK).

### 2.2. Animals and Hemolymph Samples

Mussels (*Mytilus galloprovincialis* Lam.) were purchased from an aquaculture farm (La Spezia, IT), transferred to the laboratory, and acclimatized in tanks containing aerated artificial seawater (ASW) (1 L/animal), 35 ppt, at 16 ± 1 °C for 24 h.

Hemolymph samples were extracted from the adductor muscle of the animals using a syringe, filtered with gauze, and pooled. Hemocyte monolayers were obtained as previously described [28]. Hemolymph serum (HS, i.e., hemolymph free of cells) was obtained by centrifugation of whole hemolymph samples at 900× *g* for 10 min and filtration of the supernatant through 0.22 μm filters [29].

### 2.3. In Vitro and In Vivo Exposure Conditions

All procedures for in vitro experiments were performed as previously described [26,28,29]. For each independent experiment (n = 4), the hemolymphs were sampled and pooled from 4–5 animals. Briefly, hemocyte monolayers or aliquots of the whole hemolymph, depending on the endpoint measured, were incubated at 16 °C with different types of NPs suspended in ASW or HS solution at different concentrations (10, 50, 100 µg/mL) for 30 or 60 min (according to the parameters of interest). Untreated samples were run in parallel in both media.

For in vivo assay, mussels (4 groups of 12–15 mussels each, n = 4) were exposed 24 h to different NPs at the concentration of 10 or 50 μg/L, as previously described [25]. In these conditions, NPs have been shown to rapidly affect immune parameters in vivo. Stock suspensions of PS-NPs in MQ water (1 mg/mL) were vortexed and immediately spiked in the tanks in order to reach the desired concentrations. A group of control (untreated) mussels was kept in clean ASW. Animals were not fed during the experiments. No mortality was observed in different experimental conditions. At the end of exposure, the hemolymphs were extracted, and the samples were processed as for in vitro assays.

### 2.4. Functional Immune Parameters

Hemocyte functional parameters (lysosomal membrane stability, phagocytosis, extracellular ROS production, and lysosomal enzyme release) were evaluated as previously described [26,29]. Lysosomal membrane stability (LMS) was evaluated by the NRRT (Neutral Red Retention time) assay in hemocyte monolayers incubated with a neutral red (NR) solution (final concentration 40 μg/mL) for 15 min and washed with ASW. Samples were examined under an optical microscope every 15 min until 50% of the cells showed signs of lysosomal leaking. The results are reported as percent NRRT with respect to controls.

Phagocytosis was evaluated by the uptake of neutral red-stained zymosan on hemocyte monolayers. Samples were incubated with neutral red-stained zymosan in 0.05 M TrisHCl buffer (TBS) for 1 h. Then, monolayers were washed three times with ASW, fixed with Baker’s formol calcium, and mounted in Kaiser’s glycerol gelatine medium for microscopical examination with a fluorescent microscope. For each slide, the percentage of phagocytic hemocytes was calculated from a minimum of 200 cells in triplicate.

The extracellular production of Reactive Oxygen Species (ROS) by hemocytes was measured by the reduction of cytochrome c. Aliquots of whole hemolymph (in triplicates) were incubated with 500 μL of cytochrome c solution (75 mM ferricytochrome c in TBS buffer). Samples were read for absorbance at 550 nm.

Lysozyme activity in HS samples from in vivo experiments was determined spectrophotometrically at 450 nm utilizing *Micrococcus lysodeikticus*.

Data on ROS production and lysozyme activity were corrected for total protein content, evaluated by the Bradford method using bovine serum albumin (BSA) as a standard, and expressed as percent of controls.

### 2.5. Isolation of PS_100_-S–Protein Complexes and Characterization of NP-Protein Corona by Nano-HPLC-ESI-MS/MS

All procedures for the isolation and characterization of the PS_100_-S–protein complex with *Mytilus* hemolymph serum were performed as previously reported for PS_50_-B [29,30] and details are reported in SI. Briefly, HS serum obtained from 100 mussels was dialyzed to remove excess NaCl and subsequently lyophilized. PS_100_-S were incubated with concentrated (10×) HS at the nominal concentration of 25 μg NP/mg protein/mL for 24 h at 18 °C under gentle shaking. After incubation, particle protein complexes were obtained by centrifugal isolation (see SI). After different washing steps, the pellet containing the hard corona (HC) proteins was resuspended in 0.1 mL ASW, and protein content was evaluated. Proteins were separated by 10% SDS/PAGE. Samples of PS_100_-S suspensions in ASW (25 mg/mL) and of the HC pellet resuspended in ASW at the same concentration were observed by FESEM (see SI).

### 2.6. Larval Toxicity Assay

Mussels sampled at the main spawning season (February 2021) were transferred to the laboratory and acclimatized in static tanks containing aerated artificial seawater [31], pH 7.9–8.1, 35 ppt salinity (1 L/animal), at 16 ± 1 °C. Mussels were utilized within 2 days for gamete collection as previously described [32]. At the beginning of spontaneous spawning, each individual was immediately placed in a 250 mL beaker containing 200 mL of aerated ASW until complete gamete emission. Then, mussels were removed from beakers, and sperms and eggs were sieved through 50 μm and 100 μm meshes, respectively. Egg quality (shape, size) and sperm motility were checked using an inverted microscope. Eggs were fertilized with an egg/sperm ratio of 1:10 in polystyrene 96-microwell plates (Costar, Corning Incorporate, Corning, NY, USA). After 30 min, fertilization success (n. fertilized eggs/n. total eggs × 100) was verified by microscopical observation (>85%).

Aliquots of 20 μL of 10x PS-NP suspensions in filter-sterilized ASW were added to fertilized eggs in each microwell to reach the nominal final concentrations (0–1000 μg/L) in a 200 μL volume [24]. Microplates were gently stirred for 1 min and then incubated at 18 ± 1 °C for 48 h, with a 16:8 h light/dark photoperiod. At the end of the incubation time, samples were fixed with buffered formalin (4%). The recorded endpoint was the percentage of normal D-larvae in each well with respect to the total, including malformed larvae and pre-D stages, with an acceptability of test results based on a percentage of normal D-shell stage larvae >75% in controls [31]. Four experiments were made using 6 replicate wells per condition. All larvae in each well (about 50 larvae per well) were examined by optical microscopy using an inverted Olympus IX53 microscope (Olympus, Milano, Italy) at 400× equipped with a CCD UC30 camera and a digital image acquisition software (cellSens Entry).

### 2.7. Larval Shell Formation

The deposition of larval shell components at 24 and 48 h post fertilization (pf) was visualized by Calcofluor White Fluorescent Brightener 28 (Sigma Aldrich, Lyon, France) for organic matrix and by Calcein (Sigma Aldrich, Lyon, France) for CaCO_3_ deposition, respectively, as previously described [33,34]. Larvae were imaged with a Leica SP8 Confocal Laser Scanning Microscope (CLSM-Leica, Rueil-Malmaison, France) scanning sequentially Brightfield, Calcofluor, and Calcein signals with a 0.5 µm Z-stack interval. Channels were merged and 3D rendered and rotated to measure the area (in µm^2^) of each shell component in a single valva of each larva by manual drawing using IMAGEJ software (Wayne Rasband, Bethesda, MA, USA) [33,34]. Analyses were performed on larvae obtained from 4 independent parental pairs (at least 15 individuals for each parental pair and experimental condition). Data were normalized with respect to controls for each parental pair and experimental conditions of exposure.

### 2.8. Statistics

Data are the mean ± SD of 4 independent experiments (n = 4), with each assay performed in triplicate. Data were analyzed by non-parametric Kruskal–Wallis followed by Dunn’s multiple comparisons test (* *p* ≤ 0.05) and data on the area of the organic matrix of larvae were analyzed by Mann–Whitney U test (* *p* ≤ 0.05). All statistical calculations were performed using the GraphPad Prism version 7.03 for Windows, GraphPad Software, San Diego, CA, USA.

## 3. Results

### 3.1. Particle Characterization

**Figure 1:**
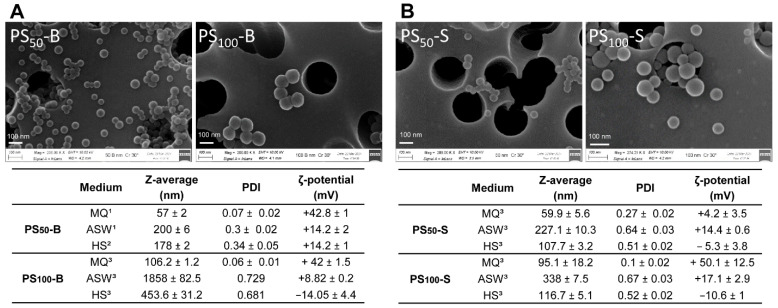
Characterization of amino-modified nanopolystyrene (PS-NH_2_) of different nominal sizes from (**A**) Bangs Laboratories, Inc. (Fishers, IN, USA); PS_50_-B and PS_100_-B; (**B**) Sigma-Aldrich (Milan, Italy); PS_50_-S and PS_100_-S. Upper panels: representative SEM images. Scale bar: 100 nm. Lower panels: physicochemical characterization and behavior in different exposure media. PDI = polydispersity index; ζ = zeta potential; MQ = Milli-Q water; ASW = artificial seawater; HS = *Mytilus* hemolymph serum. ^1^ from [28]; ^2^ from [29]; ^3^ this study.

Data on the characterization of all PS-NH_2_ are reported in Figure 1. SEM images showed a general homogenous size for primary particles PS_50_-B, PS_100_-B, and PS_50_-S, whereas PS_100_-S included also particles of smaller dimensions (Figure 1A,B).

With regard to particle behavior in different media, none of the PS-NPs showed agglomeration in MQ. However, both PS-NH_2_ from Sigma generally showed a higher PDI than Bangs’. In MQ, all particles showed a highly positive zeta potential (from about +42 to +50 mV), except for PS_50_-S (+4.2 mV). In ASW, both PS_50_-B and PS_50_-S formed small agglomerates (≈200 nm) and showed a similar zeta potential (about +14 mV). For larger particles, agglomeration was observed, in particular for PS_100_-B (1858 ± 82 nm); all particles retained a positive charge; however, values were lower than those observed in MQ, in particular for PS_100_-B. In mussel biological fluid, HS, a distinct behavior was observed for different PS-NPs. HS did not significantly affect the behavior of PS_50_-B with respect to ASW. In contrast, PS_50_-S showed a smaller agglomeration in HS than in ASW; what is more, an inversion of the zeta potential was observed (−5 mV). The decrease in agglomeration in HS with respect to ASW was more evident for larger particles, in particular for PS_100_-B. Moreover, in these conditions, both PS_100_-S and PS_100_-B showed an inversion of the zeta potential (−14.1 and −10.6 mV, respectively). Finally, in both ASW and HS, higher PDI values than in MQ were observed for all particles, indicating the higher instability of the suspensions in high ionic strength media.

### 3.2. In Vitro Effects on Mussel Hemocytes

**Figure 2:**
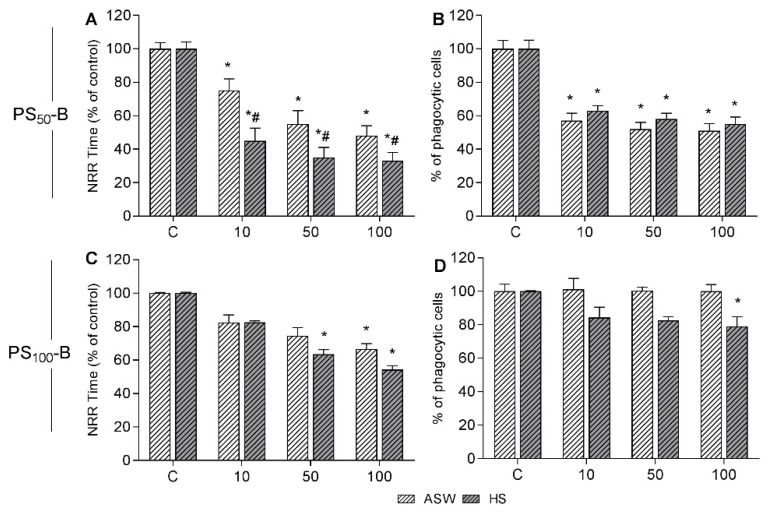
In vitro effects of PS_50_-B and PS_100_-B in hemocytes of *Mytilus galloprovincialis* suspended in either ASW (artificial seawater) or HS (hemolymph serum). Hemocytes were exposed for 30 min to different concentrations of NPs 10, 50, and 100 µg/mL. PS_50_-B: Lysosomal Membrane Stability (**A**); Phagocytic activity (**B**) PS_100_-B: Lysosomal Membrane Stability (**C**); Phagocytic activity (**D**). Data are expressed as percent of control. Statistical analyses were performed by non-parametric Kruskal–Wallis followed by the Dunn’s multiple comparisons test (*p* < 0.05). * All treatments vs. controls; # HS vs. ASW.

The short term in vitro effects of PS_50_-B and PS_100_-B in ASW and HS on mussel hemocytes were evaluated, and the data obtained for LMS and phagocytosis are reported in Figure 2. The effects on LMS were concentration dependent for both particles (Figure 2A,C). For PS_50_-B, significantly stronger effects were observed in the presence of HS. PS_100_-B was ineffective at the lowest concentration tested, showing significant effects in both media only at 100 µg/mL, which were slightly higher in HS. The hemocyte phagocytic activity was significantly reduced by exposure to PS_50_-B from 10 µg/mL in both media (about −40% with respect to controls) (Figure 2B), whereas it was scarcely affected by PS_100_-B, with significant effects only at 100 µg/mL and in the presence of HS (−20% with respect to controls) (Figure 2D).

**Figure 3:**
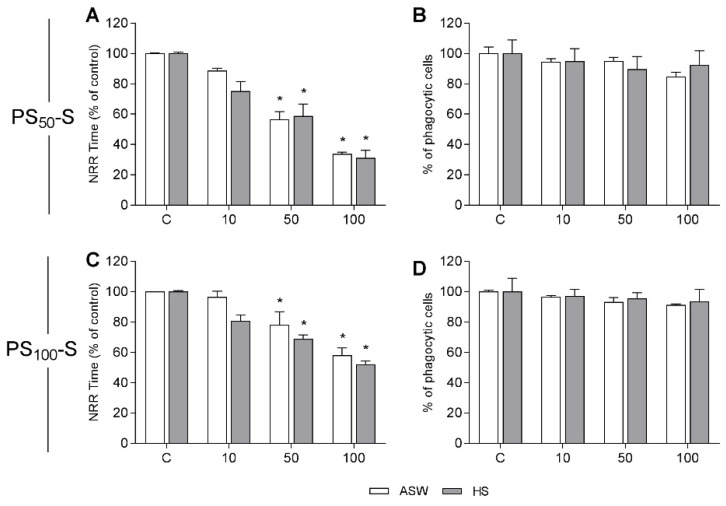
In vitro effects of PS_50_-S and PS_100_-S in hemocytes of *Mytilus galloprovincialis* suspended in either ASW (artificial seawater) or HS (hemolymph serum). PS_50_-S: Lysosomal Membrane Stability (**A**); Phagocytic activity (**B**) PS_100_-S: Lysosomal Membrane Stability (**C**); Phagocytic activity (**D**). Data are expressed as percent of control. Statistical analyses were performed by non-parametric Kruskal–Wallis followed by the Dunn’s multiple comparisons test (*p* < 0.05). * All treatments vs. controls.

Data obtained with PS_50_-S and PS_100_-S are presented in Figure 3. Particles of both sizes induced a significant decrease in LMS only from 50 μg/mL. At the highest concentration tested (100 μg/mL), PS_50_-S induced a stronger decrease (−66% with respect to control) in comparison to PS_100_-S (−40%) (Figure 3A,C). Moreover, for both PS-NH_2_, similar effects were observed in different media. The phagocytic activity was unaffected by either PS-NP at all the concentrations tested in ASW and HS (Figure 3B,D).

### 3.3. Isolation and Characterization of PS_100_-S Protein Complexes in Hemolymph Serum

Suspensions of PS_100_-S in HS were subjected to a basic protocol (centrifugation, 1D gel electrophoresis, MS) previously utilized to isolate the protein corona formed with PS_50_-B in mussel hemolymph [29,30]. A representative gel is reported in Figure S1, showing in the sample of hard corona proteins (HC) the presence of a main protein band of the apparent MW of about 20 kDa. This band was cut from the gel, trypsin digested, and analyzed by nano-HPLC-ESI-MS/MS [29,30]. The results allowed specifically identifying the main PS_100_-S–corona protein as the Putative C1q domain containing protein MgC1q44 of *M. galloprovincialis* (F0V481), with a MW of 23.6 kDa and high confidence peptides corresponding to a sequence coverage of 75.23%. Details on all the identified peptides (nine in total) related to the MgC1q44 protein are reported in Table S1.

Samples obtained by the centrifugation procedure with PS_100_-S containing the protein–corona complexes were observed by FESEM in comparison with samples of PS_100_-S suspensions in ASW, and representative images are reported in Figure S2. As shown in Figure S2A, samples in ASW formed small agglomerates, confirming data from DLS analysis. In samples containing PS_100_-S–corona complexes, individual particles were embedded in an amorphous material (Figure S2B,C).

### 3.4. In Vivo Experiments: Effects on Immune Parameters

**Figure 4:**
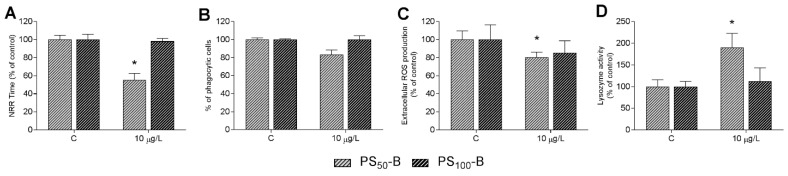
In vivo effects of PS_50_-B and PS_100_-B on hemolymph immune parameters of *Mytilus*. Mussels were exposed to both size PS-NH_2_ for 24 h at 10 µg/L. Hemocyte lysosomal membrane stability (LMS) (**A**), phagocytosis (**B**), ROS production (**C**), and serum lysozyme activity (**D**). Data are expressed as percent of control. Statistical analyses were performed by non-parametric Kruskal–Wallis followed by the Dunn’s multiple comparisons test (*p* < 0.05), * exposed vs. controls.

The effects of in vivo exposure of adult mussels to PS_50_-B and PS_100_-B (10 µg/L, 24 h) on hemolymph functional parameters are reported in Figure 4. The results show that PS_50_-B induced significant decreases in hemocyte LMS and ROS production (Figure 4A,C), (−45% and −20% vs. controls, respectively). A small although not significant decrease in phagocytosis was observed (Figure 4B). Soluble lysozyme activity was significantly increased (+90% than controls) (Figure 4D). In contrast, PS_100_-B was ineffective on all parameters tested.

**Figure 5:**
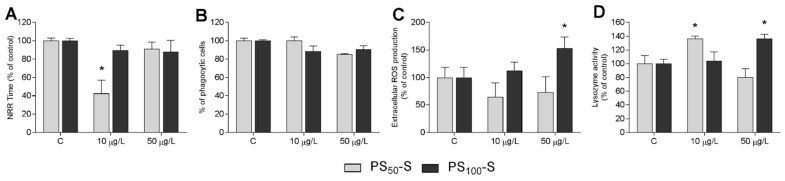
In vivo effects of PS_50_-S and PS_100_-S on hemolymph immune parameters of *Mytilus*. Adult mussels were exposed to both size PS-NH_2_ for 24 h at 10 and 50 µg/L. Hemocyte lysosomal membrane stability (LMS) (**A**), phagocytosis (**B**), ROS production (**C**), and serum lysozyme activity (**D**). Data expressed as percent of control. Statistical analyses were performed by non-parametric Kruskal–Wallis followed by the Dunn’s multiple comparisons test (* *p* < 0.05).

On the basis of the results obtained in vitro, indicating that PS-NH_2_ from Sigma were less effective than those from Bangs’, in vivo exposure of mussels was carried out at two concentrations (10 and 50 µg/L), and the results are reported in Figure 5. PS_50_-S, at the lowest concentration tested, significantly decreased LMS (−50% with respect to controls) and increased lysozyme activity (+36%), with effects similar to those recorded with PS_50_-B (Figure 5A,D), whereas no effects were observed at 50 µg/L. Phagocytosis was unaffected by either concentration (Figure 5B). A reduction in ROS production, albeit not significant, was observed at both concentrations (Figure 5C).

PS_100_-S did not affect hemocyte LMS and phagocytosis at either concentration (Figure 5A,B). However, significant increases in extracellular ROS production and lysozyme activity were observed at 50 µg/L (+52% and +37% vs. controls, respectively) (Figure 5C,D).

### 3.5. In Vivo Experiments: Effects on Early Larval Development

**Figure 6:**
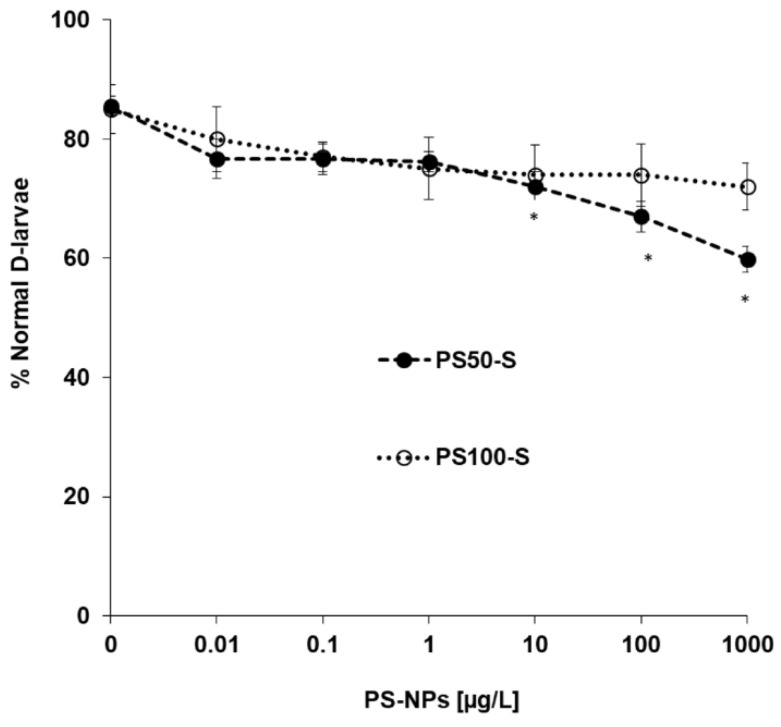
Effects of PS_50_-S and PS_100_-S on early larval development of *M. galloprovincialis*, evaluated in the 48 h embryotoxicity assay. Fertilized eggs were exposed to different concentrations of NPs in ASW (0.001–1000 μg/L). Data, reporting the percentage of normal D-shaped larvae, represent the mean ± SD of four experiments carried out in 96-multiwell plates (six replicate wells for each sample). Statistical analyses were performed by non-parametric Kruskal–Wallis followed by the Dunn’s multiple comparisons test (* *p* < 0.05).

The effects of PS_50_-S and PS_100_-S in a wide concentration range (from 0.001 to 1000 μg/L) were evaluated on early larval development in the 48 h embryotoxicity assay, as previously described for PS_50_-B [24]. The results, reporting the percentage of normally developed D-veligers at 48 hpf, are shown in Figure 6. PS_50_-B induced a concentration-dependent decrease in normal larval development that was significant from 10 μg/L, whereas PS_100_-S was ineffective. Exposure to PS_50_ essentially resulted in malformations of D-larvae.

**Figure 7:**
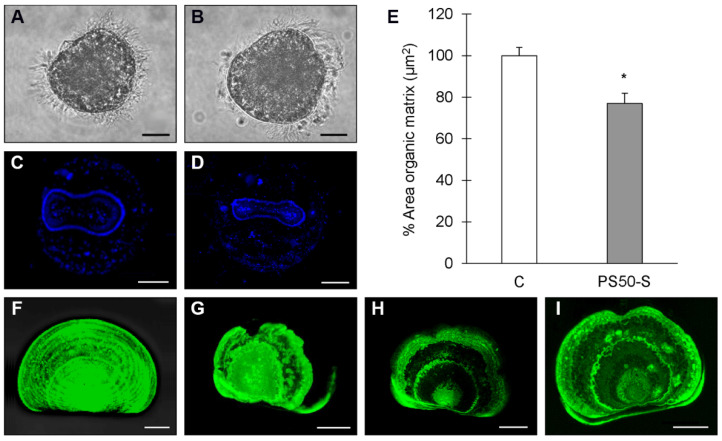
Effects of PS_50_-S on shell formation of *M. galloprovincialis* larvae at 24 (**A**–**D**) and 48 hpf (**E**,**F**) evaluated by Calcofluor/Calcein staining and confocal microscopy. Fertilized eggs were exposed to 150 μg/L PS_50_-S in ASW. 24 hpf: light microscopy images of Control (**A**) and NP-exposed larvae (**B**); calcofluor staining (blue) of the organic matrix the growing shell of Control (**C**) and NP-exposed larvae (**D**). In (**E**), the effect on PS_50_-S on the area of the organic matrix is reported (percent values with respect to control). 48 hpf: Calcein staining (green) of calcified shell. Control larvae show extensive calcification with shell accretion rings (**F**); PS_50_-S exposure results in irregular calcification and shell malformations (**G**–**I**). Scale bar: 20 µm. Statistical analyses were performed by Mann–Whitney U test (* *p* < 0.05).

The effects on larval development were further investigated from earlier stages, 24 hpf, when the processes of shell biogenesis begins [33], utilizing a single concentration of PS_50_-S (150 μg/L) chosen of the basis of previous studies carried out with PS_50_-B [24]. Deposition of the shell organic matrix and CaCO_3_ was evaluated by Calcofluor/Calcein staining and confocal microscopy. As shown in Figure 7, at 24 hpf, the larva (trocophora) is characterized by a ciliated epithelium and active movement (Figure 7A). In PS_50_-S exposed larvae, particle agglomerates around the cilia were observed, leading to apparent impairment of ciliary beating and swimming activity (Figure 7B).

At 24 hpf, in control larvae, the growing shell is represented by the saddle-shaped shell field occupied by the organic matrix (blue) (Figure 7C). In PS_50_-S-exposed samples, a significant decrease in the area of the organic matrix was observed (Figure 7D) (−23% with respect to controls, *p* < 0.05) (Figure 7E). It is worth noting that the Calcofluor signal was absent in about 30% of the larvae, indicating the absence of organic matrix deposition.

Shell calcification was evaluated at 48 hpf by Calcein staining (for CaCO_3_ deposition, green). Control shells show extensive calcification characterized by regular accretion rings (Figure 7F). Following exposure to PS_50_-S, larval malformations were associated with altered shell calcification patterns (Figure 7G–I).

## 4. Discussion

The results obtained in this work represent the first data on the comparison of different types and sources of commercial PS-NPs and their biological responses in a marine invertebrate. The results indicate that the effects were dependent on the behavior of particles of different sizes and from different sources in different exposure media. These data extend previous observations on the biological interactions of PS-NPs in the model marine bivalve *M. galloprovincialis* and provide evidence for the effects of exposure in different in vitro and in vivo settings, from the molecular to the organism level.

Although few studies utilized homemade NPs (e.g., crushed from existing nanospheres or larger plastics debris) [35,36], most data on the effects of NPs marine invertebrates have been obtained utilizing commercial PS-NPs of different sizes and surface characteristics, which were obtained from different companies, mainly from Polyscience, Inc. [12,37,38,39] and Bangs Laboratories Inc. [12,29,40,41,42]. Different particle behavior and properties were often observed in different media (changes in agglomeration state or surface charge). These variables are likely to strongly affect the biological responses observed and therefore comparisons among different studies.

To address this issue, in the present work, we compared PS-NH_2_ s of two different sizes (50 and 100 nm) and purchased by two different manufacturers. The results underline that PS-NPs of different sizes and sources have a distinct behavior in different media. Larger particles generally showed stronger agglomeration that was however distinct in ASW and HS, depending on the particle source, with less agglomeration of PS_100_-S than PS_100_-B. With regard to the surface charge, PS_100_-B and both PS-NH_2_ from Sigma showed an inversion of zeta potential in mussel HS. This was not unexpected, since changes in zeta potential were previously reported for PS_100_-S in buffer (becoming negative), while PS_50_-S remained positively charged [43]. Our data further underline the importance of distinct particle behavior in different media, and in particular in biological fluids of different species, that must be taken into account when comparing the biological effects observed in different experimental systems.

The effects of PS-NH_2_ were first evaluated on mussel hemocytes in short term in vitro tests, where the main biomarkers of cellular stress and immune function (LMS and phagocytosis) were evaluated. PS_50_-B clearly induced strong effects in both ASW and mussel HS, with a concentration-dependent disruption of LMS and a large reduction in phagocytic activity. The results are in line with the observed behavior of these NPs in both media, which was characterized by the small agglomeration and maintenance of positive zeta potential. Moreover, they confirm previous data obtained in *Mytilus* hemocytes, indicating functional cellular damage from low concentrations (5 µg/mL) accompanied by gross changes in cell morphology [26,28,29]. Interestingly, PS_100_-B induced less adverse effects on LMS and phagocytosis in both media; this could be due to the distinct behavior of larger particles, which was characterized by strong agglomeration in ASW and inversion of the zeta potential in HS, respectively.

The same trend was observed for PS-NH_2_ from Sigma on LMS, with PS_50_-S being slightly more effective than PS_100_-S. However, neither particle affected the phagocytic activity of hemocytes. In HS, the lack of responses may be partly related to the inversion of the zeta potential of both PS-NH_2_. Negatively charged NPs are generally considered less toxic than cationic particles in both mammalian cells and marine models [23,44]. However, in ASW, no significant differences in behavior were observed for Sigma PS-NH_2_ with respect to Bangs’ that could justify the smaller effects observed.

Overall, due to the large variability in particle behavior in different media, no generalities can be drawn on their effects on mussel cells, depending on the type of PS-NPs utilized and experimental factors. In the present work, we observed an important difference in surface charge and behavior among different PS-NPs in HS. However, only PS_50_-B induced stronger effects in HS than in ASW.

Researchers have now widely accepted the importance of understanding how, once within the organism, nanoparticles interact at the molecular level with cells in a physiological environment. Different types of particles associate with soluble components of biological fluids organized into a ‘protein corona’, which confers a biological identity to nanoparticles and affects their interactions with target cells [45]. We have previously investigated the protein coronas formed with different types of nanoparticles in *M. galloprovincialis* HS [30]. The results indicated that the formation of a biomolecular corona is particle-specific and that the net surface charge retained by different nanoparticles in mussel biological fluids, characterized by high ionic strength, rather than size or core composition, might be an important factor in the formation of a stable protein corona [30]. In particular, using PS_50_-B, the results identified the PutativeC1q domain containing protein (MgC1q6) as the only component of the hard protein corona formed by this type of PS-NP in HS. This protein is characterized by its affinity for cations, which is in line with the positive surface charge retained by PS_50_-B in HS [24,30]. The specific association with this immune-related protein may be responsible for the strong effects of PS_50_-B observed in mussel hemocytes.

In order to further investigate the nano-bio-interactions of PS-NPs in mussel biological fluids, we evaluated the possible formation of PS_100_-S–protein corona in HS, utilizing the same procedure previously described. PS_100_-S was chosen, since it showed the smaller effects on mussel hemocytes, and a peculiar behavior in HS, which was characterized by the absence of agglomeration and inversion of the zeta potential. This suggests strong interactions with soluble hemolymph components that may reduce the potential adverse effects of this type of NP on hemocytes. The results identified the MgC1q44 as the main hemolymph serum protein stably associated with PS_100_-S. This protein, although belonging to the wide group of MgC1q complement proteins of *M. galloprovincialis* [46], when searched in the UNIPROT database did not show any sequence identity with MgC1q6 or other proteins of *M. galloprovincialis* and only a 50% identity with MCOR_19868 of *M. coruscus*. Although at present, the physiological function of this protein is unknown, it may be responsible for the characteristic behavior of PS_100_-S in HS. The distinct composition of the protein corona formed with PS_50_-B and P_100_-S further support the specificity of nano-bio-interactions occurring in mussel physiological medium, resulting in a distinct outcome of the biological response.

The effects of PS-NH_2_ were also investigated at the whole organism level, evaluating different functional immune responses after short in vivo exposure of adult mussels (24 h). In these conditions, NPs have been shown to induce changes in immune parameters [25,47]. Experiments were initially run with PS-NH_2_ from Bangs at 10 µg/L, which is in line with previous works performed with PS_50_-B [25,47]. As in in vitro experiments, PS_50_-B induced significant effects on LMS, ROS production, and lysozyme activity, whereas PS_100_-B was ineffective. This absence of effects could be related to the strong agglomeration observed in ASW, which may limit the uptake of these particles and consequent transfer to the hemolymph.

For in vivo exposure to PS_50_-S and PS_100_-S, which showed smaller effects in in vitro experiments, a higher concentration (50 µg/L) was also tested. Even though PS_50_-S was less effective than PS_50_-B at the same concentration of 10 µg/L, LMS and lysozyme activity were significantly affected. In contrast, PS_100_-S induced extracellular ROS and lysozyme release only at higher concentrations; it was also more effective than PS_100_-B, which was probably due to its small agglomeration in ASW and consequent higher uptake. Overall, the results obtained in vivo strongly support the link between the differences in the behavior of different PS-NH_2_ in ASW, according to their size and source, and the biological effects observed.

Previous data showed that PS_50_-B affected early larval development; the effects were partly ascribed to dysregulation of the transcription of genes involved in early shell formation [24]. Here, we investigated the effects of PS-NPs from Sigma: the results indicate that PS_50_-S affected the development of 48 hpf larvae from 10 μg/L. Although at higher concentrations, the effects were smaller than those previously observed with PS_50_-B, here, we show that PS_50_-S (150 μg/L) significantly affected the mechanisms involved in shell biogenesis. PS_50_-S decreased the deposition of the organic shell matrix from 24 hpf and of calcification at 48 hpf, resulting in altered larval phenotypes. In contrast, PS_100_-S did not affect larval development at any concentration tested. Similar results were obtained with PS_100_-B (Balbi, unpublished observations). Taken together, the results further support the hypothesis that in mussels, early larval development can represent a sensitive target for certain types of PS-NPs. In oysters, 50 and 100 nm PS-NPs from different sources induced a decrease in fertilization rate [48], and agglomerates were attached to the sperm and the jelly coating of eggs, impairing their mobility and fertilization success [49]. However, the effects were observed at much higher concentrations (mg/L) than those utilized in the present study (μg/L).

## 5. Conclusions

Overall, the results add information on the nano-bio-interactions occurring between NPs and possible target species [19,20,21] in the marine environment. Moreover, our data underline that care must be taken in comparing and interpreting the results obtained on the effects of different types of commercial NPs for the same polymer. As to the environmental relevance of the result obtained, as already underlined in the introduction, the experimental approach utilized in the present study has obvious limitations in terms of types of NPs used, due to the absence of environmental NP samples for toxicity testing; however, at present, this applies to most available studies on the biological impact of NPs. With regard to exposure conditions, predicted environmental concentrations are estimated from 1 pg to 15 μg/L for ≈50 nm plastic particles [50], and concentrations up to 15 μg/L in seawater have been considered environmentally realistic for studies on marine bivalves [51]. The results here obtained in in vivo experiments indicate that with 50 nm NPs, significant effects on both immune parameters and larval development could be observed from 10 μg/L, therefore at environmentally relevant concentrations. Further studies are needed utilizing non-homogeneous nanoplastics such as those obtained from weathering or fragmentation of larger plastic debris, fibres, and from different polymers.

## Data Availability

Not applicable.

## Supplementary Materials

The following are available online at https://www.mdpi.com/article/10.3390/nano11123291/s1, Figure S1: Representative gel of the separation of the PS_100_-S protein complexes from HS of *M. galloprovincialis* proteins by SDS-PAGE and staining with Coomassie Brilliant Blue. Table S1. Identification on HC peptides by Nano-HPLC-ESI-MS/MS. Figure S2: FESEM analysis of PS_100_-S suspensions I ASW an HS.
