# Supplementary material for: Comparison of Different Commercial Nanopolystyrenes: Behavior in Exposure Media, Effects on Immune Function and Early Larval Development in the Model Bivalve Mytilus galloprovincialis
Source: Nanomaterials (Basel). 2021 Dec 4;11(12):3291. doi: 10.3390/nano11123291 (PMC8705110; doi:10.3390/nano11123291)
Supplement: Supplementary file 1 [file nanomaterials-11-03291-s001.zip › nanomaterials-1483792-supplementary.pdf]

nanomaterials

Article

Comparison of Different Commercial Nanopolystyrenes: Behavior in Exposure Media, Effects on Immune Function and Early Larval Development in the Model Bivalve *Mytilus galloprovincialis*

Manon Auguste [1,*], Teresa Balbi [1], Angelica Miglioli [1], Stefano Alberti [2], Sonja Prandi [3], Riccardo Narizzano [3], Annalisa Salis [4], Gianluca Damonte [4] and Laura Canesi [1]

1 Department of Environmental, Earth, and Life Sciences (DISTAV), University of Genoa, 16136 Genoa, Italy; teresa.balbi@unige.it (T.B.); angelica.miglioli@obs-vlfr.fr (A.M.); laura.canesi@unige.it (L.C.)
2 Department of Chemistry and Industrial Chemistry (DICCI), University of Genoa, 16136 Genoa, Italy; stefano.alberti@edu.unige.it
3 Department Regional Laboratory, Sector Organic Chemistry and Physical Analysis, ARPAL, 16149 Genoa, Italy; sonja.prandi@arpal.liguria.it (S.P.); riccardo.narizzano@arpal.liguria.it (R.N.)
4 Department of Experimental Medicine (DIMES), University of Genoa, 16136 Genoa, Italy; Annalisa.Salis@unige.it (A.S.); gianluca.damonte@unige.it (G.D.)
* Correspondence: manon.auguste@edu.unige.it

Methods

Isolation of PS_{100}-S-protein complexes and Nano -HPLC-ESI-MS/MS

All procedures were as previously described [1,2].

Hemolymph serum (HS) obtained from about 60 mussels was filter sterilized, dialysed overnight at 16 °C against MilliQ water with 10 kDa cutoff tubes, subsequently lyophilized and kept at -80 °C. Samples were resuspended in sterilized ASW and protein content was adjusted to 10 mg/mL. PS_{100}-S were incubated with HS at the nominal particle concentration of 25 µg/mg protein for 24 h at 16 °C on a rocking platform. All experiments were carried out at least twice in duplicates. After incubation, particle–protein complexes were recovered by centrifugal isolation. Samples were centrifuged at 17,000x g for 75 min at 4 °C. The supernatant was stored at -80 °C (SN) and the pellet was re-suspended in ASW, transferred to a new vial, and centrifuged again to pellet the particle-protein complexes. This washing procedure, used for removing unbound and loosely bound proteins from NPs, was repeated three times, to obtain W1, W2 and W3 samples. The pellet, containing the hard corona (HC) proteins, was re-suspended in 0.1 mL ASW and protein content was evaluated, as well as in SN, W1, W2, W3 samples. W3 samples did not contain any detectable amount of proteins. Samples were added with SDS- sample buffer and boiled for 5 min. Proteins (25 µg) were separated by 10% SDS/PAGE [1,2].

Bands of interest were cut from the gel and destained, reduced, alkylated and digested with trypsin. Samples were analysed by HPLC-MS/MS using an Ultimate 3000 nano-HPLC system (managed by CHROMELEON software, version 6.70SP2a, LC Packings, Amsterdam, NL) connected to a Hybrid Quadrupole-Orbitrap mass spectrometer (QExactive, Thermo Scientific). Trypsinized peptide pellets were resuspended immediately before analysis and were first loaded from the sample loop onto a trapping column (Acclaim PepMap C18; 2 cm × 100 µm × 5 µm, 100 Å- Thermo Scientific) using the loading solvent (95-5% ACN/H_2O+ 0.05% trifluoroacetic acid) at a flow rate of 5 µL/min for 5 min. The trapping column was then switched in-line with the separation column and the peptides were eluted with increasing organic solvent at a flow rate of 300 nL/min. The separations were carried out at 35° C using an Easy spray column (15cm x 75µm PepMap C18 3 µm Thermo Scientific) applying a linear gradient of solution B (95-5% ACN/H_2O+ 0.08%

formic acid) from 4% to 95% in 55 minutes. All analyses were carried out in the positive ion mode. Single MS survey scans were performed in the Orbitrap, recording a mass window between 395 and 2000 m/z using a maximal ion injection time of 100 ms. The resolution was set to 70,000 and the automatic gain control was set to $3x10^6$ ions. The experiments were done in data-dependent acquisition mode with alternating MS and MS/MS experiments. The minimum MS signal to obtain MS/MS was set to 500 ions, with the most prominent ion signal selected for MS/MS using an isolation window of 2 Da. The m/z values of signals already selected for MS/MS were put on an exclusion list for 5 s using dynamic exclusion. In all cases, one micro-scan was recorded. CID was done with a target value of 5000 ions, a maximal ion injection time of 50 ms, normalized collision energy of 35%. A maximum of 10 MS/MS experiments/MS scan were performed. Raw MS files were processed with the Thermo Scientific Proteome Discoverer software version 1.4. Peak list files were searched by the SEQUEST search engine against Uniprot database for "Bivalvia", containing both forward and reverse protein sequences. The resulting peptide hits were filtered for a maximum 1% FDR (false discovery rate) using percolator, the peptide mass deviation was set to 10 ppm and a minimum of six amino acids per identified peptide were required. The database search parameters were: mass tolerance precursor 20 ppm, mass tolerance fragment CID 0.8 Da with dynamic modification of deamidation (N, Q), oxidation (M) and static modification of alkylation with IAM (C). For all searches, the option trypsin with two missed cleavages was selected.

Field emission scanning electron microscopy

Samples of PS_{100}-S suspensions in ASW (25 mg/mL) were pelleted by centrifugation and resuspended in MilliQ water. In parallel, samples of the pellet obtained by the centrifugation procedure applied to the PS-NP suspension in HS (25 mg/mg protein/mL) to separate the NP corona proteins, containing PS-NP –protein corona complexes (HC), were resuspended in 1mL of MilliQ water. Both samples were vortexed and two drops of each suspension were placed on a lacey carbon holder and left to dry in air without coating. Samples were observed by FESEM (Zeiss, Sigma 300, Jena, Germany) operating at 10 kV.

Results

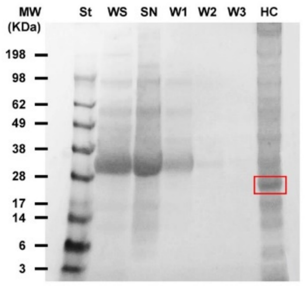

Figure S1. Representative gel of the separation of the PS_{100}-S protein complexes from HS of *M. galloprovincialis* proteins by SDS-PAGE and staining with Coomassie Brilliant Blue. Lanes : St = molecular weight standards: WS = whole serum sample in the absence of NPs (before all the purification and washing steps): SN = supernatant after the first centrifugation of the PS100-S suspension in HS; W1, W2, W3 = samples corresponding to the three washing steps. HC = long lived, hard corona proteins. The red square indicates the position of band excised for tandem mass spectrometry analysis.

After in gel reduction, carbamidomethylation and tryptic digestion of the gel band at 23kDa showed the presence of various proteins, including the Putative C1q domain containing protein MgC1q44 OS=*Mytilus galloprovincialis* (F0V481). While the Sequest scores of the other proteins were low (<15), F0V481 was identified with a 71 Sequest score

through the characterization of nine high confidence peptides, corresponding to a sequence coverage of 75.23%. A table reporting some parameters as confidence, post translational modifications (PTMs) and mass accuracy ΔM [ppm] relative to the peptides used to identify the MgC1q44 protein are reported. To note that peptides number 6 and 7 only differ only for the presence of a deamidated asparagine; peptides number 1 and 2 differ for the presence of one or none oxidated methionine, respectively; peptides 3, 5 and 9 represent fragments containing one missed cleavage.

Table S1. Identification on HC peptides by *Nano -HPLC-ESI-MS/MS.*

F0V481	Putative C1q domain containing protein MgC1q44 OS=Mytilus galloprovincialis GN=MgC1q44 PE=2 SV=1 - [F0V481_MYTGA]	SEQUEST SCORE 71				
#	Confidence	Sequence	Modifications	MH+ [Da]	ΔM [ppm]	# Missed Cleavages
1	High	LGGGNVAFSSYmTEASANSQSASLTT-GSTIK	M12(Oxidation)	3053,46549	-1,27	0
2	High	LGGGNVAFSSYMTEASANSQSASLTT-GSTIK		3037,48001	4,15	0
3	High	FDRTETNYGnGYSR	N10(Deamidated)	1680,73899	1,79	1
4	High	QAIGGLQTR		943,54198	1,96	0
5	High	GDDAcSTGFVIEYVSKGTEVYLR	C5(Carbamidomethyl)	2566,24729	1,52	1
6	High	GnSVIGLLHADTETR	N2(Deamidated)	1583,81980	4,41	0
7	High	GNSVIGLLHADTETR		1582,83550	3,75	0
8	High	TETNYGnGYSR	N7(Deamidated)	1262,53850	2,21	0
9	High	VLKAEVDR	M12(Oxidation)	929,55045	5,98	1

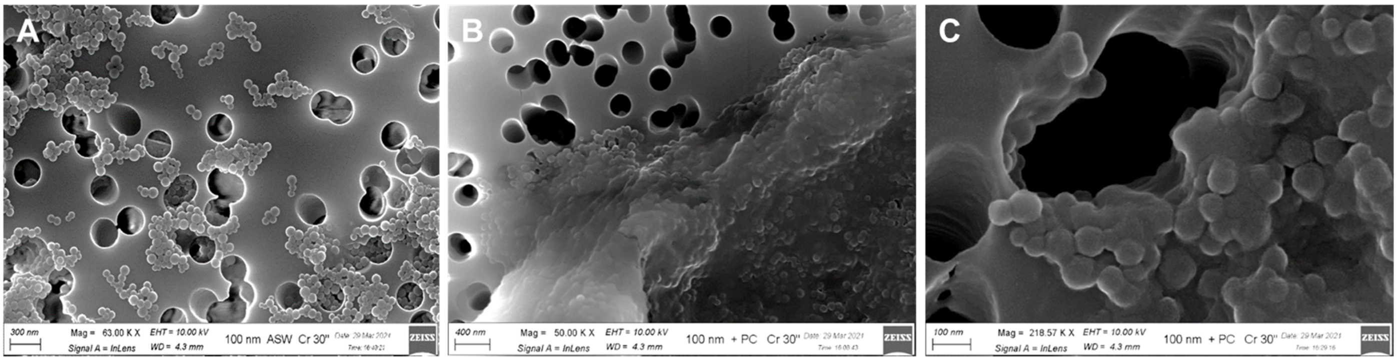

Figure S2. – FESEM analysis of PS_{100}-S suspensions in ASW (A) (25 mg/mL) and in samples of Table 100. S -proteins (25 mg/mg prot/mL) obtained after the centrifugation steps (B,C).

References

1. Canesi, L.; Ciacci, C.; Fabbri, R.; Balbi, T.; Salis, A.; Damonte, G.; Cortese, K.; Caratto, V.; Monopoli, M.P.; Dawson, K.; et al. Interactions of Cationic Polystyrene Nanoparticles with Marine Bivalve Hemocytes in a Physiological Environment: Role of Soluble Hemolymph Proteins. *Environ Res* **2016**, *150*, 73–81, doi:10.1016/j.envres.2016.05.045.
2. Canesi, L.; Balbi, T.; Fabbri, R.; Salis, A.; Damonte, G.; Volland, M.; Blasco, J. Biomolecular Coronas in Invertebrate Species: Implications in the Environmental Impact of Nanoparticles. *NanoImpact* **2017**, *8*, 89–98, doi:10.1016/j.impact.2017.08.001.
